# Evaluation of Serum Adiponectin Concentrations Among Drug Abusers on Methadone Maintenance Treatment

**DOI:** 10.5812/ijhrba.14021

**Published:** 2013-12-24

**Authors:** Farzaneh Montazerifar, Mansour Karajibani, Kobra Lashkaripour, Maryam Yousefi

**Affiliations:** 1 Pregnancy Health Research Center, Zahedan University of Medical Sciences, Zahedan, IR Iran; 2 Health Promotion Research Center, Zahedan University of Medical Sciences, Zahedan, IR Iran; 3 Department of Nutrition, School of Medicine, Zahedan University of Medical Sciences, Zahedan, IR Iran; 4 Department of Psychiatry and Psychology, Baharan Psychatric Hospital, Zahedan University of Medical Sciences, Zahedan, IR Iran

**Keywords:** Adiponectin, Drug Users, Methadone

## Abstract

**Background:**

Adiponectin, an adipocyte-derived protein, modulates a number of metabolic processes. Methadone maintenance treatment (MMT) changes the level of hormones produced by adipose tissue in addicts. However, current data remains contradictory.

**Objectives:**

The aim of this study was to evaluate the effect of MMT on serum adiponectin levels in drug addicts.

**Materials and Methods:**

Twenty-five drug abusers with a mean age of 37.4 ± 8.7 years were referred to the Baharan Hospital, Zahedan, and 22 healthy age-matched control subjects with a mean age of 35 ± 9.5 years were enrolled in the study. Addicts were treated with methadone at (40 to 120 mg/d) for six months. Measurement of anthropometric parameters, serum adiponectin, and biochemical parameter levels, were assessed in the addicts, before and after six months of MMT, but only once in the healthy controls.

**Results:**

The mean basal serum adiponectin level was not significantly lower in the drug abuser group compared to the healthy subjects (P > 0.05). After six months of MMT, the mean serum adiponectin level of the drug addicts was not significantly different from their mean baseline level or that of the healthy subjects (P > 0.05). However, the mean baseline serum adiponectin level was significantly lower in overweight/obese addicts when compared to underweight patients and healthy individuals (P < 0.001). After six months of MMT, the mean level of serum adiponectin increased significantly in the underweight subjects compared to the normal weight and overweight/obese subjects (P < 0.0001) and the control group (P < 0.001). Adiponectin concentration was correlated inversely with body mass index and positively correlated with waist circumference and serum high-density lipoprotein levels.

**Conclusions:**

This study showed that MMT did not markedly alter the concentration of serum adiponectin in drug abusers. However, in regard to the variations in the serum lipid profiles and anthropometric parameters, the findings indicated that low concentrations of serum adiponectin might play a role in the pathogenesis of obesity and other metabolic abnormalities. Thus, more long-term studies with larger sample sizes are recommended.

## 1. Background

Drug abuse and its related complications are one of the most important public health problems in many parts of the world (1, 2). Nutritional deficits including; weight loss and changes in dietary patterns of addicts, have been reported in previous studies (1, 3, 4). Methadone maintenance treatment (MMT) is used in the treatment of heroin and other opiate abusers (1, 5), and it may be affected by improvements in their nutritional status (4, 5), and the many hormonal disturbances observed among drug abusers (1). However, previous studies have mentioned that opiate abusers lost their appetite after they started treatment, which might be related to psychiatric co-morbidities including depression and personality disorders (5). It has been reported that MMT changes the levels of adipose tissue-derived hormones in addicts (1, 6). Adipose tissue has an important role to play in the regulation of energy intake and expenditure, and the metabolism of lipids and carbohydrates (7). Adiponectin is an adipose tissue-specific protein (7-10) with anti-atherogenic and anti-inflammatory properties (7-9, 11-13). Moreover, circulating concentrations of adiponectin might be an important marker for some other aspects of a person’s health.

Several components of metabolic abnormalities have been related to low levels of adiponectin such as; the risk of obesity especially intra-abdominal body fat distribution (7, 14), insulin resistance, type 2 diabetes mellitus, and dyslipidemia (7, 15), low high-density lipoprotein (HDL) levels, high levels of low-density lipoproteins (LDL), apolipoprotein B, and triglycerides (TG) (7, 16). A variety of factors affect adiponectin levels such as; sex, age, race, and ethnicity (17, 18).

## 2. Objectives

There is limited data about serum adiponectin concentrations in addicts and its significance in drug abuse patients in MMT has not been investigated sufficiently. The aim of this study was to describe the effect of MMT on serum adiponectin levels among drug addicts.

## 3. Materials and Methods

This study was carried out on 25 drug abusers (20 males and 5 females) with a mean age of 37.4 ± 8.7 years and a body mass index (BMI) of 23.2 ± 7.6 kg/m^2 ^who were referred to the Baharan Hospital, Zahedan, capital of Sistan and Baluchestan Province, southeastern Iran. These patients were compared with 22 healthy age-matched control subjects (12 males and 10 females) with a mean age of 35 ± 9.5 years, and a mean BMI of 22 ± 1.9 kg/m^2^.

Weight and height were measured in a standing position. BMI was calculated as weight in kilograms divided by the square of their height in meters (kg/m^2^). The categorization of BMI was based on the guidelines of the Center for Disease Control and Prevention (19, 20). BMIs ≤ 18.5 were considered as underweight, BMIs between 18.5 and 24.9 were normal, and BMIs ≥ 25 were categorized as overweight/obese.

Waist circumference (WC) was measured at the top of the hip bone. WCs > 102 cm in men or > 88 cm in women were considered as abdominal obesity (20). Blood pressure was measured in a sitting position. 

Blood samples were taken after an overnight fast. Serum adiponectin levels were determined by a commercial enzyme-linked immunosorbent assay kit (BioVendor; Cat No: RD 191001100, USA). Samples were immediately frozen at -70°C until required.

Fasting blood sugar (FBS), total serum cholesterol, LDL, HDL, and TG concentrations, were measured with a colorimetric method (Technicon RA-1000 system, USA).

Then, the drug addicts were treated with methadone at a dosage of between 40 to 120 mg/d, according to a clinic-based psychiatric diagnosis. After six months, anthropometric measurements and the previously mentioned blood tests were repeated for the drug addicts, while the healthy controls were assessed only once. The study protocol was approved by the Ethics Committee of Zahedan University of Medical Sciences, Iran. Informed consent was obtained from all patients and healthy control subjects.

### 3.1. Statistical Analysis

The statistical analysis was performed using SPSS (version 15.0) software for Windows (SPSS Inc., Chicago, Ill). All data were normally distributed and were reported as mean (SD). Comparisons between the patients and control group were performed with one-way analysis of variance (ANOVA) followed by a Tukey’s test where appropriate.

The differences among the groups, based on their BMI status, were analyzed with 2-way ANOVA and for repeated measures, analysis of variance using a Bonferroni test. Two groups of participants with and without abdominal obesity were compared using a Student’s t-test. The correlations between the different quantitative variables were determined by a Pearson correlation test. P values less than 0.05 were considered significant.

## 4. Results

Demographic and clinical characteristics of the subjects are shown in Table 1. While there was no significant difference between drug abusers before MMT and control subjects regarding their age, weight, and BMI, the WC was significantly higher in the addicts compared to controls (P < 0.01). After six months of MMT, the mean values of BMI and WC were dramatically elevated in the addicts when compared with healthy controls (P < 0.01). MMT did not significantly affect blood pressure. The serum levels of adiponectin and biochemical parameters of the subjects are described in Table 2. There was no significant difference between serum adiponectin levels before (1.58 ± 0.9 μg/mL) and after six months of MMT (1.67 ± 1 μg/mL) and these levels remained lower relative to the control group (2 ± 1.4 μg/mL, P > 0.05). 

**Table 1.** Demographic and Clinical Characteristics of Drug Abusers Before and After Methadone Maintenance Treatment and Controls ^a^

	Before MMT^b^ (n = 25)	After MMT (n = 25)	Controls (n = 22)
**Sex, No. (%)**			
Male	20 (80)	20 (80)	12 (54.5)
Female	5 (20)	5 (20)	10 (45.5)
**Age, mean ± SD, y**	37.4 ± 8.7	37.4 ± 8.7	35 ± 9.5
**Weight, mean ± SD, kg**	62.6 ± 9.6	68 ± 12.6^c^	60.5 ± 4.9
**BMI^b^** **, mean ± SD, kg/m²**	23.2 ± 7.6	28 ± 9.5^c^	22 ± 1.9
**WC^b^** **, mean ± SD, cm**			
Male	87.4 ± 12.3	93 ± 10.8^c^	82.2 ± 8.3
Female	97.4 ± 10.8	107 ± 9.6^c^	77.2 ± 12.1
**Blood pressure, mean ± SD, mmHg**			
Systolic	11.6 ± 1.1	11.3 ± 0.66	11.5 ± 0.96
Diastolic	7.6 ± 0.5	7.3 ± 0.48	7.3 ± 0.65	

^a^ One-way ANOVA followed by Tukey’s test were used to analyze the data.

^b^ Abbreviations: BMI, body mass index; MMT, methadone maintenance treatment; WC, waist circumference.

^c^ P < 0.01 versus before MMT; P < 0.01 versus control group.

**Table 2.** Serum Levels of Adiponectin and Other Biochemical Parameters of Heroin Addicts Before and After Methadone Maintenance Treatment and Controls ^a^

	Before MMT^b^	After MMT	Controls
**Serum adiponectin, mean ± SD (range), ng/mL**	1.58 ± 0.9 (0.79-4.43)	1.67 ± 1 (0.52-4.62)	2 ± 1.4 (0.53-9.8)
**FBS** ^**b**^ **, mean ± SD, mg/dL**	90.7 ± 13.1	84.5 ± 14	86.8 ± 10.3
**Serum TC** ^**b**^ **, mean ± SD, mg/dL**	177.7 ± 38.8	177. 3 ± 36.5	170 ± 39.2
**Serum LDL** ^**b**^ **, mean ± SD, mg/dL**	99 ± 32.1	114.6 ± 35.6	110.2 ± 36.6
**Serum HDL** ^**b**^ **, mean ± SD, mg/dL**	56.7 ± 11.9	51.8 ± 11.7	56.9 ± 8.3
**Serum TG** ^**b**^ **, mean ± SD, mg/dL**	160.6 ± 104^c^	171 ± 116^c,d^	110.5 ± 80.2

^a^ One-way ANOVA followed by Tukey’s test were used to analyze the data.

^b^ Abbreviations: FBS, fasting blood sugar; HDL, high-density lipoprotein; LDL, low-density lipoprotein; MMT, methadone maintenance treatment; TC, total cholesterol; TG, triglyceride.

^c^ P < 0.01 (versus control group)

^d^ P < 0.01 (versus before MMT)

No changes were found after MMT in the biochemical parameters of addicts with the exception of serum triglyceride levels, which increased significantly when compared with the levels before MMT and the control group (P < 0.01). While the serum LDL level also tended to increase, the difference was not significant.

As shown in Table 3, the basal serum adiponectin level was significantly lower in overweight/obese addicts when compared to normal and underweight subjects, and the control group (P < 0.001). After six months of MMT, the mean serum level of adiponectin increased significantly in the underweight subjects compared to normal and overweight/obese subjects (P < 0.0001), and the control group (P < 0.001). 

A significant negative correlation was found between adiponectin levels with BMI (r = -0.15, P = 0.02), and a significant positive correlation with serum HDL levels (r = 0.78, P = 0.001) was found after MMT. In addition, a positive correlation between serum adiponectin levels with WC was found both before (r = 0.43, P = 0.003) and after (r = 0.52, P = 0.02) MMT. No significant correlation was found between serum adiponectin levels and the other study variables.

**Table 3.** Levels of Serum Adiponectin in Heroin Addicts Before and After Methadone Maintenance Treatment and Controls According to Anthropometric Parameters ^a^

	Before MMT^b^	After MMT	Controls
**BMI^b^ status, mean ± SD**			
Underweight	1.64 ± 1.4^c^	2.8 ± 1.4	-
Normal	1.9 ± 0.8	1.4 ± 0.49^e^	1.9 ± 0.9
Overweight/Obese	1.1 ± 0.58 ^d,e^	1.4 ± 0.44^e^	-
**WC^b^, mean ± SD, cm**			
< 88	1.65 ± 0.99	1.85 ± 1.1	2 ± 1.1
> 88	1.6 ± 0.83	1.5 ± 1.12	1.65 ± 0.88

^a^ Two-way ANOVA followed by Bonferroni test were used to analyze the data.

^b^ Abbreviations: BMI, body mass index; MMT, methadone maintenance treatment; WC, waist circumference.

^c^ P < 0.0001 (versus normal and overweight after MMT)

^d^ P < 0.001 (versus normal and underweight before MMT)

^e^ P < 0.001 (versus control group)

## 5. Discussion

The results of this study showed that the mean level of serum adiponectin in drug addicts before and after MMT was lower when compared with the control group. The serum level of adiponectin was significantly lower in the overweight/obese addicts compared with the underweight subjects and control group, at baseline. However, a significant increase was found in the mean level of serum adiponectin in underweight subjects when compared with normal and overweight/obese subjects and the control group. It had previously been reported that serum adiponectin concentrations normally increase in lean subjects and decrease in obese subjects (1, 6, 21, 22).

A prior study conducted by Cnop et al. (23), showed that the serum adiponectin level decreased in obese individuals. A negative correlation was found between the plasma adiponectin level and visceral fat which was significantly stronger than that of subcutaneous fat (23). It has been suggested that a possible explanation for the decline in serum adiponectin concentrations observed in obesity might be that adiponectin secretion from visceral adipose tissue is higher than that of large triglyceride-filled visceral adipocytes. Furthermore, omental adipocytes that were isolated from subcutaneous fat produced more adiponectin than adipocytes (24).

The physiological role of adiponectin has not been fully established. However, it has been proposed that low concentrations of serum adiponectin observed in obese subjects (10, 21, 22, 25, 26) might contribute to the development of atherosclerosis and cardiovascular diseases (8, 12, 13, 16, 17). There is evidence that adiponectin affects the regulation of both lipid and carbohydrate metabolism. Low levels of adiponectin are associated with high levels of serum LDL and triglycerides (16). Elevated serum levels of LDL are one of the major risk factors for the development of coronary artery disease (9). In the current study, concentrations of serum lipid were not pathologically elevated in addicts in comparison with those of the control group, and only baseline serum triglyceride levels of the drug addicts were found to be higher. Furthermore, serum LDL levels also tended to increase after MMT, but the difference was not significant (P > 0.05).

Previous cross-sectional studies have indicated that circulating adiponectin concentrations are negatively correlated with serum triglycerides and positively correlated with HDL concentrations (9, 27, 28).

Based on the criteria of the International Diabetes Federation in 2005 (17, 28), and the national cholesterol education program-adult treatment panel III (29), the presence of at least three to four major abnormalities including; obesity, especially abdominal obesity, hypertriglyceridemia, low HDL hypercholesterolemia, hypertension, and elevated FBS, could be considered as metabolic syndrome. In the present study, insignificant changes in serum levels of adiponectin and lipid profile were found after six months of MMT. Variations of anthropometric parameters and an inverse correlation were observed between serum adiponectin levels with BMI and a positive correlation with WC and serum HDL. However, the findings showed that low concentrations of serum adiponectin, when compared with those of the control group, might lead to metabolic abnormalities.

Our study had some limitations such as; small sample size and relatively short intervention time. Furthermore, because central adiposity can affect serum adiponectin concentrations, the subjects were matched based on their WC, but not their BMI.

This study showed that MMT did not markedly alter the concentration of serum adiponectin in drug abusers. However, taking into account the variations of serum lipid profile and anthropometric parameters, the findings indicate that serum adiponectin levels might play a role in the pathogenesis of obesity and other metabolic abnormalities. Thus, more long-term studies with larger numbers of subjects are recommended.
